# GrowCAD: bioinspired mathematical design for additive manufacturing

**DOI:** 10.1098/rsos.242229

**Published:** 2025-09-24

**Authors:** Nasim Mahmoodi, Galane Jingxi Luo, Rosemary Julia Dyson, Lauren Elizabeth Jane Thomas-Seale

**Affiliations:** ^1^ School of Engineering, University of Birmingham, Birmingham, UK; ^2^ School of Mathematics, University of Birmingham, Birmingham, UK

**Keywords:** engineering design, additive manufacturing, 3D printing, bioinspired, mathematical biology

## Abstract

While the socioeconomic and environmental benefits of additive manufacturing (AM) are acknowledged, design for AM remains a perpetual challenge in the wider implementation of the technique. Design in the context of AM is an interconnected and broad topic. It encompasses not only function and form, but also how geometry is represented digitally, the associated software and human problem-solving capabilities within the geometric opportunities and constraints. This research focuses on enhancing human knowledge and creativity within the bounds of an ever-evolving design space, encompassing digital and human capabilities. A bioinspired methodology is introduced, drawing an analogy between plant growth and the layer-by-layer AM process. This results in the development of a novel length-polar-projection coordinate system, and the associated algebraic definition of centre lines and cross-sections. This mathematical representation of geometry forms the foundation of the design framework, GrowCAD^TM^. Retaining the algebraic format of the geometry enables a manufacturability analysis, parametric editability and computer-aided design compatibility. The research is validated through qualitative analysis of the shape fidelity and efficiency, the ability to detect non-manufacturable geometry, the end-to-end functionality and the printability of the successful geometries. The simplicity and intuitive nature of GrowCAD^TM^ offer a method by which to enhance the engineer’s knowledge and creativity.

## 1. Introduction

The potential economic, social and environmental benefits of additive manufacturing (AM) are widely renowned [1–3]. The rapid development of a broad spectrum of hardware, encompassing a diverse selection of materials, has seen swift implementation in both research and commercial environments over the last two decades. Yet, one perpetuated challenge that has seen a comparably slower development and poses a limitation to industrial uptake, is design [4].

As a discipline, it is a challenge to define the boundaries of design. The term can refer to the design of the functionality of a mechanical system, through to the economic impact of choosing suppliers for materials or components. Efficient design requires comprehensive knowledge of functionality, materials and manufacturing, and also the complementary breadth of knowledge to understand the impact of these design choices [5,6]. In the context of design for AM (DfAM), there are several additional challenges [7]. To design the geometry of a part requires knowledge of the capabilities of manufacturing in the context of materials and function, in terms of both opportunities and limitations [8]. The rapid development of AM hardware means that manufacturability, associated with each platform and compatible materials, is evolving. In essence, what geometry is possible and what is not is constantly changing in line with developments in hardware capabilities, and knowledge becomes rapidly obsolete. Furthermore, AM can form complex geometry above and beyond those associated with subtractive manufacturing; as such it requires enhanced creativity within the bounds of the design space [9].

The lynchpin of design is computer-aided design (CAD). While several approaches to optimize and automate design exist (discussed in §2.1), CAD and the ability to parametrically edit geometry in the context of a history or series of previous edits remain central to the process. Yet, CAD is heavily based on the extrusion and subtraction of primitive solids, a process that mirrors the formation of shapes using machining processes [10]. This contrasts with AM, which forms three-dimensional (3D) models by joining material layer by layer [11,12]. The purpose of this research is to develop a digital method of creating geometry, which is a better reflection of the physical process of AM, and to encourage the user to ‘think additively’. This is a broad and complicated problem in AM: to increase knowledge and creativity within the bounds of an evolving design space, bridging between digital capabilities and the human ability to learn and problem-solve.

Byrne *et al*. [13] explore the concept of bioinspired principles with respect to manufacturing technologies; encompassing bioinspired and broadening the definition to include bio-integration and bio-intelligent [13]. The study concluded that ‘biologicalisation’ represents a ground-breaking frontier in manufacturing technologies and systems, through digitalization and industry 4.0 [13]. Indeed, the study by Kalogerakis *et al*. [14] found a positive correlation between analogical distance and the novelty of a solution [14]. As such, the research presented in this article adopts a bioinspired approach to explore similarities between AM and growth, inspired by plant biology and developed between mathematics and engineering.

The aim of this research is to develop a CAD-compatible mathematical design approach for 3D printing, to enable more efficient generation of manufacturable curved geometry. The novel contribution to research knowledge is the mathematical framework, presented in §3. The method of implementation with parametric CAD, and onwards to physical 3D printing, is outlined in §4. The mathematical framework and implementation method are validated against three measures: shape fidelity and efficiency in comparison to CAD, manufacturability, and end-to-end functionality (§5). With relation to the wider purpose of the research, additional reflections around the usability and user-interface of the technique are discussed.

## 2. Background

### 2.1. Design for additive manufacturing

When critically reflecting on a geometric design process, it is important to consider the problem from a holistic perspective; geometry is not created in isolation but exists within a wider system of design processes (conceptual design through to production specifications) which interact with people, across digital platforms and at every stage. The importance of a broad perspective has been further magnified in the DfAM process, because during AM, the material is created in tandem with the form [15], and as such DfAM exists at the interface of design and manufacturing, where the resultant geometry is influenced not just by CAD but materials, manufacturing processes, parameters and post-processing [16].

There are many review papers that discuss the broad range and nuanced details of challenges that fall under the definition of DfAM [17,18]. This article will focus on summarizing the barriers associated with knowledge propagation and software to set the context of the research. When design engineers do not have first-hand knowledge of the capabilities and limitations of a manufacturing technique, then education and communication across the design–manufacturing interface remain the only routes to transfer relevant knowledge. Yet, inefficiencies in human communication across the design–manufacturing interface, that is, the exchange of knowledge between design and production engineers, are a long-standing and perpetuating problem [19–21]. The lag of education behind development is recognized as a major problem inhibiting wider adoption of the technology [22]. These issues are further exacerbated by psychological inertia (sustaining a method of thinking that prevents innovation), which is prevalent in engineering [23].

To enable comprehensive manufacturing knowledge during the design phase, the primary focus of industry and research has been digital approaches, which either assist in adhering to AM geometric constraints and/or exploit the design flexibility of the approach. Examples of widely used commercial approaches are topology optimization (TO) and generative design. The concept of TO is to optimize the geometry of a structure towards a certain parameter, such as mass or stiffness, through the removal or placement of material [24]. While TO has good industrial uptake, with methodologies such as evolutionary structural optimization [25] and level set methods [26] driving innovation for decades, crucial questions about benchmarking and standardization remain unanswered [27,28]. Generative design, implemented using Fusion 360 (Autodesk, San Francisco, CA, USA), opens up a much wider design space, based upon pre-defined volume constraints and utilizes artificial intelligence to find a range of possible solutions to satisfy an optimization function [29]. While these techniques, and others, exploit the geometric freedom associated with AM, the automated approach does not enrich the knowledge of the design engineer. Instead, such ‘black-box’ techniques, which take inputs and create outputs without giving the user knowledge of the methodology, create a dependency on software. This in turn creates a social and economic barrier to its use and, therefore, a barrier to AM as a technology.

As an alternative, free-form representation of surfaces can be enabled by non-uniform rational B-splines (NURBS) modelling, which gives more flexibility than Boolean operations. However, NURBS raises challenges for the design engineer in the definition of detailed features and combining with other geometry [30]. Surface modelling may require specialized training and more experience than traditional solid modelling. Therefore, the efficient modelling of complex products through NURBS may be subject to the same knowledge propagation issues as seen in AM [15].

### 2.2. A mathematical bioinspired approach

The opportunity that exists when engineering mimics form and functions seen in nature has been acknowledged in systems [31,32], materials [33,34] and less commonly manufacturing [9,13]. Geometries that have been computationally optimized (e.g. function or mass) and are manufacturable using AM are often described as looking like organic or natural structures. The capacity to create hierarchical structures and responsive materials, characteristics that are intrinsically found in nature, means that leveraging AM to enable the formation of bioinspired materials, geometries and systems has been explored through many lenses.

Utilizing bioinspiration in manufacturing processes presents a ground-breaking opportunity to advance the field. Bryne *et al*. [13] define ‘Biologicalisation in Manufacturing’ as ‘The use and integration of biological and bioinspired principles, materials, functions, structures and resources for intelligent and sustainable manufacturing technologies and systems with the aim of achieving their full potential’ [13]. Essentially, bioinspiration is a vehicle towards increased efficiency and sustainability. However, the translation of such biological analogies to improve AM technology or design processes has remained largely un-investigated. Thomas-Seale *et al*. [35] draw reference to the similarities between *in utero* growth, cell by cell and the incremental fusion of material in AM [35]; however, this study does not progress the research beyond design theory. Ren *et al*. [36] explores the parallels between four-dimensional (4D) printing (where an object can change shape in response to an external stimulus) and the actuation mechanisms of plants [36] but do not progress the research onto the mechanics of growth or actuation. In the current study, the analogy between AM and plant growth is progressed to a mathematical definition by expressing both systems in the language of differential geometry, which describes curves and surfaces in 3D space. In developing this approach, an opportunity is created to upskill the engineer to understand the complexities of curves and surface formation in the context of manufacturing constraints for AM.

**Figure 1.**
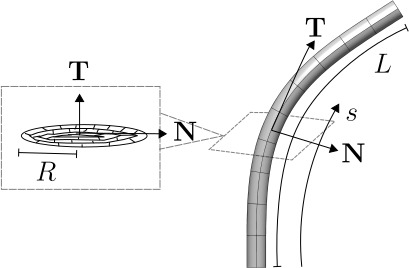
A parametrically defined upscaled structure which will twist and bend in response to changes in the cross-sectional properties, where T, N, *L*, *R* and *s* represent unit tangent to the curve, unit normal to the curve, axial length, cross-sectional radii and arclength parameter, respectively (modified from [37]).

Consider the definition of a plant root in figure 1, modified from [37]. The geometry of a root may be expressed as a series of cross-sections stacked along a construction line, parametrized by arc length *s*. The geometric design of forms where cross-sectional radii are small compared with axial length (R≪L) can be represented in the same way. The total arc length will increase as the part ‘grows’. In mathematical biology, the root geometry is the background arena in which physical effects such as force balance and conservation of angular momentum take place, and writing the corresponding equations in a suitable coordinate system provides a model of plant growth. In §3, a coordinate system suited to the growing part will be described, leading to mathematical expressions for the curves and surfaces that constitute the part’s geometry.

## 3. Mathematical development

### 3.1. Length polar projection

For this article, a construction line is a smooth curve in 3D space making an acute angle with the upward vertical at every point along the curve. Mathematically specifying such a curve typically entails writing down three functions of some arclength parameter s, for example, some x(s), y(s) and z(s) in Cartesian coordinates or some ρ(s), φ(s) and z(s) in cylindrical polar coordinates. As s varies between two values, typically normalized to 0 and 1, the values of the three functions provide the 3D coordinates of points on the curve between two ends.

A simpler, more intuitive system of curve specification has been developed, called the length-polar-projection (LPP) system, whereby only two functions and a number are sufficient to define a 3D curve uniquely. Letting T(s) denote the unit tangent to the curve, measured along increasing s (from 0 to 1), then the number is the total length L of the curve, and the two functions are a *polar angle* $\theta_{z}(s)$, from the positive *z*-axis to T(s), and a *projection angle* $\theta_{x}(s)$, from the positive *x*-axis to the *x–y* projection of T(s). It has been shown that as long as $0\leq \theta_{z}\left(s\right)<\pi /2$ for all s, meaning the curve makes an acute polar angle at every point, then the LPP inputs (L, $\theta_{z}(s)$, $\theta_{x}(s)$) are uniquely translatable to the Cartesian system by the following equations (a detailed derivation can be found in [38]):


(3.1) $$x\left(s\right)=L\int_{0}^{s}\sin\theta_{z}\left(t\right)\cos\theta_{x}\left(t\right)\;dt,$$

(3.2) $$y\left(s\right)=L\int_{0}^{s}\sin\theta_{z}\left(t\right)\sin\theta_{x}\left(t\right)\;dt,$$

(3.3) $$z\left(s\right)=L\int_{0}^{s}\cos\theta_{z}\left(t\right)\;dt.$$

The point s=0 has been set to $\left(x\left(0\right),y\left(0\right),z(0))=(0,0,0\right)$ without loss of generality. The practical simplicity of the LPP system has been demonstrated in [38].

An important quantity associated with the construction line is its local curvature, κ(s), defined by


(3.4) $$\kappa \left(s\right)=\left|\frac{d\;T\;\left(s\right)}{ds}\right|.$$

The role that κ plays in defining 3D designs will be described in §3.2.

### 3.2. Three-dimensional designs through specifications of cross-sections

Once a construction line has been defined, it remains to specify a number of cross-sections perpendicular to the construction line; and then connecting the cross-sections in a consistent manner yields a design in 3D. The designer may choose the number of cross-sections that they require, which would be a decision linked to the desired part complexity and accuracy. For solid parts, it is sufficient to specify the boundaries of the cross-sections, each boundary being a closed planar curve. To begin, a number of equally spaced ‘construction points’ are defined along the construction line, and ‘construction planes’ through the construction points are defined using a ‘tilt angle’ ψ, with ψ=0 giving a plane perpendicular to the construction line. Every construction point acts as the origin of a two-dimensional (2D) coordinate system in its construction plane. In each plane, a cross-sectional boundary is defined mathematically by the radial position *R* as a function of some angle θ, once the ‘reference direction’ in which θ=0 has been specified. It is vital that this reference direction does not change too abruptly from one construction plane to the next, otherwise practical difficulties would arise when connecting multiple cross-sections. To that end, a ‘rotation angle’ ρ in each plane is used to customize the reference direction. Thus, for each construction point, the two numbers ψ and ρ and the function R(θ) are sufficient to determine a cross-sectional boundary uniquely.

The default reference direction may be defined in two different ways, which we designate the ‘TNB’ and ‘XYZ’ methods, respectively. In each case, the construction plane is at first perpendicular to the construction line, the default direction together with ρ and R(θ) then enable a boundary curve to be defined within the plane, and finally ψ is applied to tilt the boundary curve.

#### 3.2.1. TNB alignment

On any smooth curve in 3D, every point s with unit tangent vector T(s) and non-zero curvature κ(s) gives rise to a unit normal vector N(s) defined by


(3.5) $$\frac{d\;T\left(s\right)\;}{ds}=\kappa \left(s\right)N\left(s\right).$$

By definition, N is perpendicular to T.

**Figure 2.**
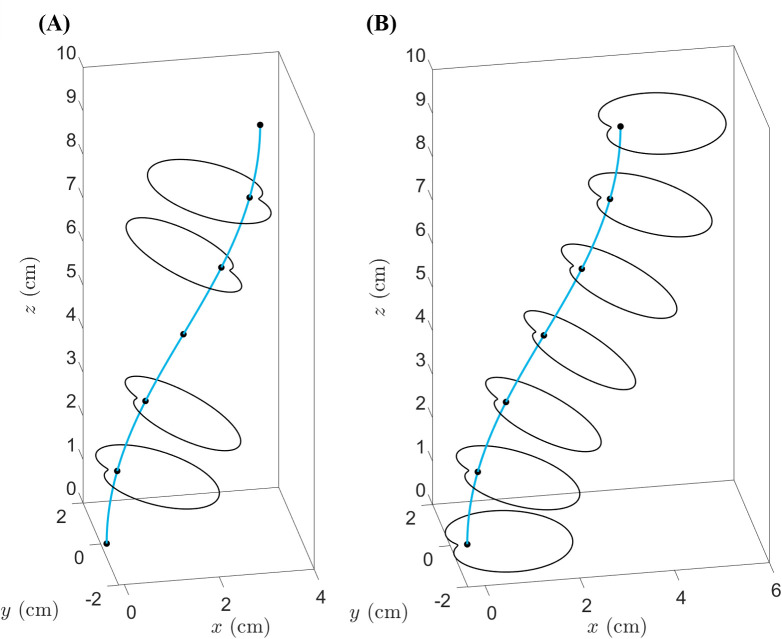
Example construction lines and planes with or without the TNB algorithm to ensure that cross-sections are well defined at every point and do not reorient abruptly. (A) Using the standard normal vector as the reference direction leads to undefined and/or abruptly reorienting cross-sections. On this construction line, there are three construction points where the local curvature is zero. At these points, the normal vector is undefined; hence, the absence of cross-sections. Moreover, as this line passes through its mid-point, the normal vector flips its direction; hence the abrupt reorientation (by 180°) of cross-sections. (B) Using the TNB alignment algorithm, all reference directions are well defined and do not reorient abruptly from one construction point to the next.

Using N(s) as the reference direction in the construction plane at s presents two possible problems. First, at points where the construction line’s curvature κ=0, the vector N is undefined; second, as the construction line passes through a point of zero curvature, the direction of N may flip (see figure 2A). The following procedure is used to fix both problems at once.

Suppose the construction line, denoted by α, is not a straight-line segment. Assume that κ can only vanish at isolated points. GrowCAD^TM^ locates all such points and measures the smallest s-gap between consecutive ones, denoting it by Δs. Let the number of construction points (which must include the end points of α) be m+1, so that 1/m is the s-gap between consecutive construction points. A large positive integer n is chosen such that 1/n<Δs and n is a multiple of m. The construction line is discretized into n equal parts, so that the n+1 discretization points are $s_{i}=i/n$ for i=0,1,2,…,n. By choice, the construction points are a subset of ${\{s}_{i}\}$, and the construction line cannot have vanishing curvature at two consecutive discretization points. GrowCAD^TM^ finds the smallest j such that $\kappa (s_{j})\neq 0$, and uses equation (3.5) to calculate $N(s_{j})$. If $N(s_{j})$ has a positive *z*-component, it is rotated by 180°. For i=j+1,j+2,…,n, GrowCAD^TM^ calculates $N(s_{i})$, skipping any point where $\kappa \left(s_{i}\right)=0$. At each step i, if the x–y projections of $N(s_{i})$ and $N(s_{i-1})$ differ by more than 90°, $N(s_{i})$ is rotated by 180°. After the i=n step, for each i where $s_{i}$ was skipped, including any i<j, $N(s_{i})$ is defined as the linear interpolation of the N vectors at the two nearest neighbours of $s_{i}$. In practice, the condition κ=0 should be replaced by κ<ε where ε is some suitably small precision (e.g. 1 arcsecond per mm). This algorithm produces n+1 unit normal vectors, including m+1 vectors that originate from the construction points. These m+1 vectors are used to define the reference directions in the construction planes. The reference directions defined in this manner do not reorient abruptly from point to point (see figure 2B).

If the construction line α is a straight-line segment, then $\kappa \left(s\right)=0$ for all s and so N(s) is not defined anywhere by equation (3.5). GrowCAD^TM^ then defines N(s) as the unit vector which is perpendicular to T(s), has an x–y projection that is parallel to the x-axis and a non-positive z-component. Such a projection is always well defined because the polar angle of α is always acute. A subset of the N(s) vectors are then used as the reference directions in the construction planes.

#### 3.2.2. XYZ alignment

**Figure 3.**
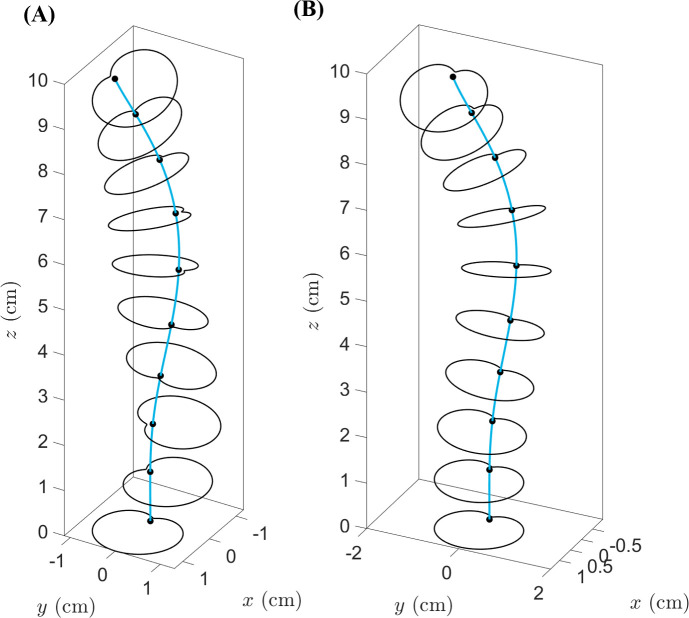
Example construction lines and planes demonstrating the TNB and XYZ alignment algorithms. Given a construction line that bends out of the plane (by twisting around the *z*-axis), the two alignment methods described in §3.2 yield different default behaviours. (A) The TNB method provides cross-sections that rotate with the twisting of the construction line, by fixing the direction of alignment with respect to an internal frame of reference which reorients along the line. (B) The XYZ method fixes the direction of alignment with respect to the external Cartesian frame of reference.

As an alternative to the TNB alignment method, the reference direction in each construction plane can simply be defined by the unit normal vector whose x–y projection is parallel to the x-axis and whose z-component is non-positive. This method, designated ‘XYZ alignment’, does not require the local curvature and is therefore computationally simpler than TNB. However, TNB provides a more ‘natural’ alignment, in the sense that, when the construction line bends out of the plane, the reference directions provided by TNB rotate accordingly, whereas those provided by XYZ do not rotate (see figure 3).

Once the boundary curves are specified in 3D space, GrowCAD^TM^ chooses N equally spaced points on each curve, where N is the smallest power of 2 such that the spacing is less than some prescribed precision, and labels the points $r_{1},\ldots ,r_{N}$ with $r_{1}$ given by the reference direction. A MATLAB extrapolation method (the ‘makima’ function) then fits a curve through all points labelled $r_{1}$, and the process is repeated for $r_{2},\ldots ,r_{N}$. The result is a discretized representation, or surface mesh, of the outer boundary of the desired geometric design. Examples of such outputs from GrowCAD^TM^ are presented in §5.1.

### 3.3. Manufacturing constraints

GrowCAD^TM^ assesses two basic manufacturing constraints, overhang angle and wall thickness, with respect to the discretized geometry. Both are local properties of the geometry, requiring analysis of neighbouring points in the mesh. Note that only solid parts are considered for this article, and therefore a mesh of the outer boundary is sufficient for the analysis.

#### 3.3.1. Overhang angle

The overhang constraint is tested point-by-point throughout the boundary mesh. The objective is to determine whether each point would be supported by material if printed. To satisfy the overhang constraint, supporting material need not be directly below the point; it suffices to find material within a cone below the point, and the overhang limit (e.g. 45°) determines the opening angle of the cone (e.g. 90°). To achieve this, the boundary mesh is organized into cross-sections, labelled $C_{0},\ldots ,C_{n}$ with $C_{0}$ on the base plate. For every point $p=(p_{x},p_{y},p_{z})$ in the mesh except for $C_{0}$, GrowCAD^TM^ identifies the largest K such that all points in $C_{K}$ have z-coordinates less than $p_{z}$; or, if such a K does not exist, then K is set to 0. GrowCAD^TM^ then constructs a series of cones with their apex at p, opening downwards with half-angle β and heights $h_{1},\ldots ,h_{N}$, where β is the user-defined overhang limit and $h_{1},\ldots ,h_{N}$ the vertical distances to each point in $C_{K}$. The base of a cone with height $h_{i}$ is a horizontal disc of radius $h_{i}\tan\beta$ centred at $\left(p_{x},p_{y},p_{z}-h_{i}\right)$. If the base of any such cone intersects the boundary surface of the part, then the point p is considered to be supported by some portion of the part lower than p; otherwise, GrowCAD^TM^ flags the point p as possibly exceeding the overhang limit and advises the user to revise the design or risk losing printability.

#### 3.3.2. Wall thickness

GrowCAD^TM^ analyses two types of wall thickness which we designate ‘perpendicular’ and ‘centre’, respectively. For this article, all cross-sectional boundary curves designed by GrowCAD^TM^ are smooth, which means that at every spline point q on a boundary curve there exists a well-defined direction perpendicular to the curve. For each q, GrowCAD^TM^ chooses the point p such that the line pq is closest to being perpendicular to the line between the left and right neighbours of q. The distance between p and q is used as a proxy for the perpendicular thickness at q and compared with a prescribed threshold. Any perpendicular thickness smaller than the threshold incurs a warning to the user. Moreover, 0.5 times the same threshold is compared with the distance between q and the centroid of all the spline points and a warning is incurred if the centre-thickness is too small.

## 4. Implementation methodology

### 4.1. Computational

The methodology described in §3 can be implemented through any programming language. In this study, it was implemented using MATLAB 2022a (MathWorks, Natick, MA, USA). Electronic supplementary material, S1, provides an excerpt of the source code GrowCAD.m, which details the implementation of the three key algorithms described in this article: the LPP system for defining construction lines (§3.1), the specification of non-axisymmetric cross-sections with two distinct alignment modes (§3.2), and the local analysis of manufacturability in terms of overhang angle and wall thickness (§3.3). As a piece of standalone software, GrowCAD.m^1^ includes a bespoke intuitive GUI allowing access to the full suite of GrowCAD^TM^ algorithms and example geometries (see appendix A, figure 7). This includes a library of geometries, which the user can select from a drop-down list, and then either use directly or fine-tune according to need. Examples of construction line shapes include a wave and a corkscrew, and examples of cross-sections, including elliptical, heart- and star-shaped boundaries. Finally, the geometry is reconstructed in CAD using the point cloud, which describes the construction line and cross-sections and is then exported from MATLAB.

As described in §4.1, GrowCAD^TM^ produces outputs that include a construction line and cross-sections defined by the outer boundaries and presented as a point cloud. The GrowCAD^TM^ software necessitates an interface with CAD software to transform these data into a 3D geometry and prepare it for manufacturing. Fusion 360 was selected for this purpose, as a widely used CAD software.

The generated point cloud is imported into Fusion 360 and processed using a Python application programming interface (API) (electronic supplementary material, S2 and S3) as follows:

(1) Determining the sketch plane for individual cross-sections using three points from that section.
(2) Defining the outer cross-section by fitting the spline curve and connecting the points to form a closed curve.
(3) Creating a 3D structure using guided or non-guided loft to connect the cross-sections.

**Figure 4.**
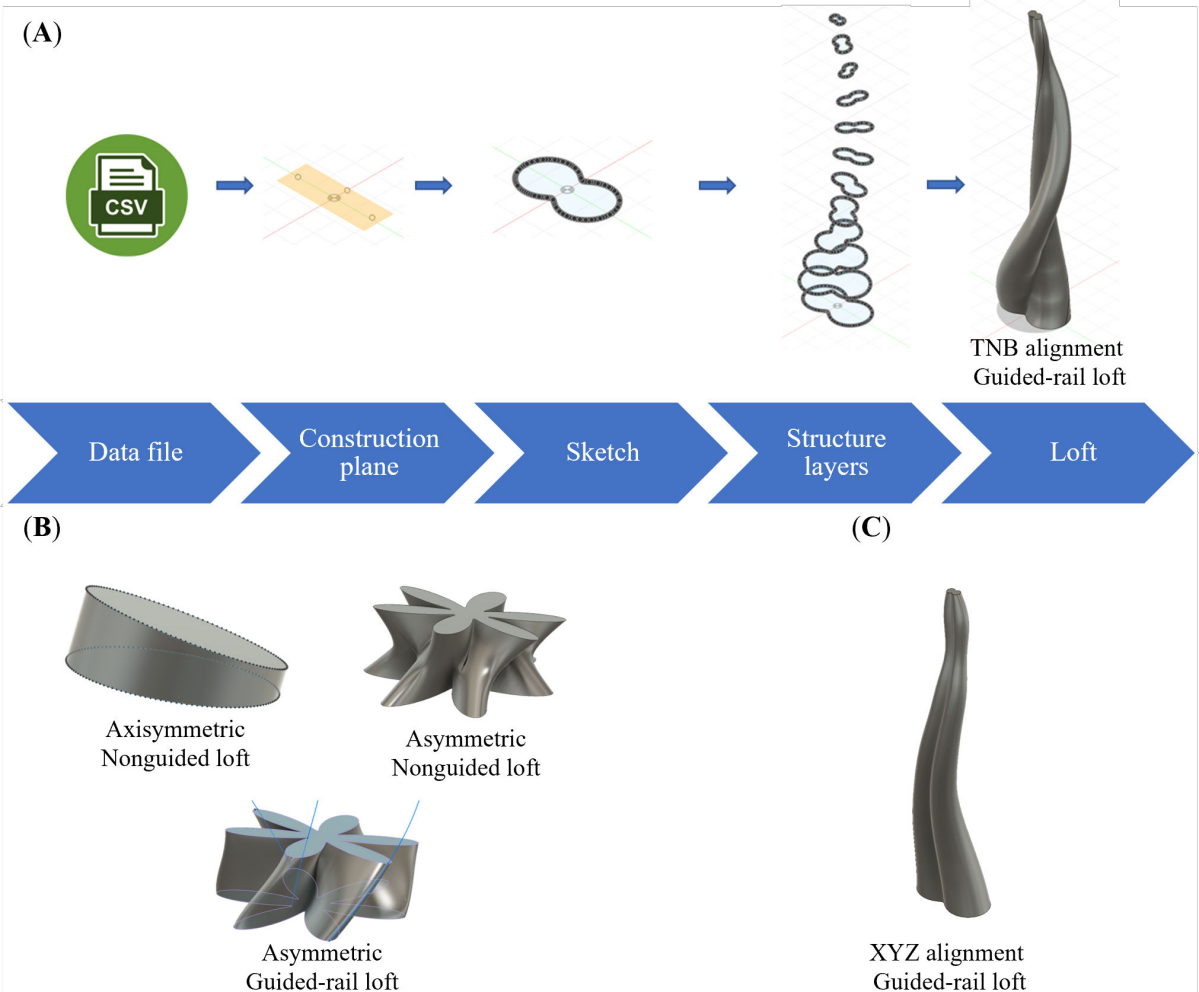
(A) Interface workflow of GrowCAD^TM^ with Fusion 360. (B) Non-guided and guided lofted 3D structure examples. (C) 3D construction using XYZ alignment with guided-rail loft.

The developed interface can convert the data to a 3D solid CAD model using guided and non-guided loft functions. While the non-guided loft is suitable for axisymmetric geometries, for asymmetric models, the incorporation of guided rails within the lofting process ensures a more precise 3D model, as shown in figure 4B. GrowCAD^TM^ allows the 3D geometry to be created using TNB and XYZ alignment as discussed in §§3.2.1 and 3.2.2. In the TNB alignment, the twisting of the line changes the normal, and subsequently the orientation of the boundary (figure 4A). In contrast, the XYZ alignment fixes the direction of alignment with respect to the external Cartesian frame of reference (figure 4C).

### 4.2. Experimental

The mathematical approach (§3) and computational implementation (§4.1) exist independently of the physical 3D printing. The approach can be made compatible with any 3D printing manufacturing technique, platform or material by adapting the manufacturing constraints, as described in §§3.3.1 and 3.3.2.

The purpose of the experimental work presented in this article is to demonstrate the end-to-end implementation of the GrowCAD^TM^ approach; from the mathematics through to the 3D printing. A Creality Ender-3 FDM 3D printer (Shenzhen Creality 3D Technology Co. Ltd, China) was used, combined with blue polylactic acid (PLA) filament (Polymaker, RS Components, UK) of 1.75 mm diameter. Computer-aided manufacturing (CAM) was set up using Ultimaker Cura V5.11 (Ultimaker BV, The Netherlands) using the Creality Ender-3 set-up options and the manufacturing parameters outlined in table 1.

**Table 1.** Manufacturing parameters (Creality Ender-3).

printing parameters	value
nozzle temperature (°C)	200
build plate temperature (°C)	50
printing speed (mm s^−1^)	60
infill pattern	grid
infill density (%)	20
nozzle diameter (mm)	0.4
layer height (mm)	0.2
wall thickness (mm)	0.8

Therefore, the manufacturing parameters (shown in table 1) were not optimized for the geometry or material. The geometric manufacturability constraints (§3.3) were defined as those widely accepted for fused deposition modelling (FDM): an overhang angle of 45° and a minimum wall thickness of 2 mm. It should be noted that the geometric constraints for manufacturing used in this study were conservative and (anecdotally) the Creality Ender-3 is known to be able to print beyond these thresholds.

## 5. Validation

To validate the hypothesis that the algebraic definition relative to the LPP coordinate system will enable a more efficient approach to generating curved geometry within user-specified manufacturing constraints, while retaining the ability to edit the geometry in CAD, a series of characteristics need to be identified:

(1) Shape fidelity and efficiency relative to the equivalent CAD methodology.
(2) The ability to detect when a proposed geometry falls outside of the bounds of the user-specified manufacturing constraints.
(3) The end-to-end functionality with CAD, CAM and FDM 3D printing.

The design of experiments is outlined in table 2: the functionality relative to the manufacturing constraints (P1), the end-to-end compatibility with CAD, CAM and FDM 3D printing (P2) and to compare with the equivalent process using CAD (P3). Experiment P3 will adopt a qualitative analysis of the computational process of constructing a geometry in GrowCAD^TM^ and then the equivalent process in CAD, to assess the difference in methodology. To conduct P3 the ‘Equation Driven Curve’ add-in was required [39]. The ‘Equation Driven Curve’ add-in in Fusion 360 enables the definition of curves through parametric equations. P1 will computationally demonstrate the ability of the approach to identify geometry that breaches the manufacturing constraints of overhang angle and wall thickness. P2 will computationally and experimentally demonstrate the capacity of the technique, and its compatibility with associated software. Where applicable, additional results will be presented in appendix B.

**Table 2.** Design of experiments: parameters of the construction line and cross-sections.

			length polar projection				first cross-section	last cross-section		
	premise	summary	*L*	*θ_z_*(*s*)	*θ_x_*(*s*)	*P*	radius	radius	rotation	tilt
P1-1-1	constraint: overhang angle	in-plane curves below threshold	12	45s	0	12	1	1	N/A	N/A
P1-1-2		in-plane curves above threshold	12	60s	0	12	1	1	N/A	N/A
P1-1-3		out-of-plane curve below threshold	12	12	360s	12	1	1	N/A	N/A
P1-1-4		out-of-plane curve above threshold	12	60	360s	12	0.3	0.3	N/A	N/A
P1-2-1	constraint: wall thickness	below threshold	6	30sin⁡(3πs)	0	12	0.2(2+cos⁡(3θ))	$0.2\left(2+\cos\left(3\theta \right)\right)$	N/A	N/A
P1-2-2		above threshold	6	30sin⁡(3πs)	0	12	$0.2\left(1.3+\cos\left(3\theta \right)\right)$	$0.2\left(1.3+\cos\left(3\theta \right)\right)$	N/A	N/A
P2-1-1	construction line	off-axis single helix construction line	8	40s	360s	12	1.5	1.5	N/A	N/A
P2-1-2		out-of-plane 2D wave construction line	8	$20\sin\left(3\pi s\right)$	100s	12	$1.8\sqrt{0.8\cos{\left(\theta \right)}^{2}+0.1\sin{\left(\theta \right)}^{2}}$	$1.8\sqrt{{0.8\cos\left(\theta \right)}^{2}+{0.1\sin\left(\theta \right)}^{2}}$	N/A	N/A
P2-1-3		on-axis double helix construction line	8	30	720s	12	$0.6\sqrt{0.8\cos{\left(\theta \right)}^{2}+0.4\sin{\left(\theta \right)}^{2}}$	$0.6\sqrt{{0.8\cos\left(\theta \right)}^{2}+{0.4\sin\left(\theta \right)}^{2}}$	N/A	N/A
P2-2-1	cross-sections	size	8	10	360s	12	$1\sqrt{0.8\cos{\left(\theta \right)}^{2}+0.1\sin{\left(\theta \right)}^{2}}$	$0.4\sqrt{0.8\cos{\left(\theta \right)}^{2}+0.1\sin{\left(\theta \right)}^{2}}$	N/A	N/A
P2-2-2		shape	8	10	360s	6	0.6(1.5+cos⁡(5θ))	$0.6\left(1.5+\cos\left(3\theta \right)\right)$	−170	N/A
P2-2-3		rotation	8	$25\sin\left(3\pi s\right)$	0	12	$0.5\left(1.2+\cos\left(\theta \right)\right)$	$0.5\left(1.2+\cos\left(\theta \right)\right)$	360	N/A
P2-2-4		size, shape and orientation	8	0	0	5	$0.4\left(3+\cos\left(6\theta \right)\right)$	$0.3\left(1.5+\cos\left(3\theta \right)\right)$	varied	varied
P3	shape fidelity	on-axis single helix, 5pt star to 3pt star cross-sections	8	10	360s	12	$0.4(1.5+\cos\left(6\theta \right))$	$0.4(1.5+\cos\left(3\theta \right))$	N/A	N/A

*L*, *θ_z_*(*s), θ_x_*(*s*) and *P* represent total length of the curve (cm), polar angle (degree), projection angle (degree) and number of used construction points, respectively.

### 5.1. Shape fidelity and efficiency

**Figure 5.**
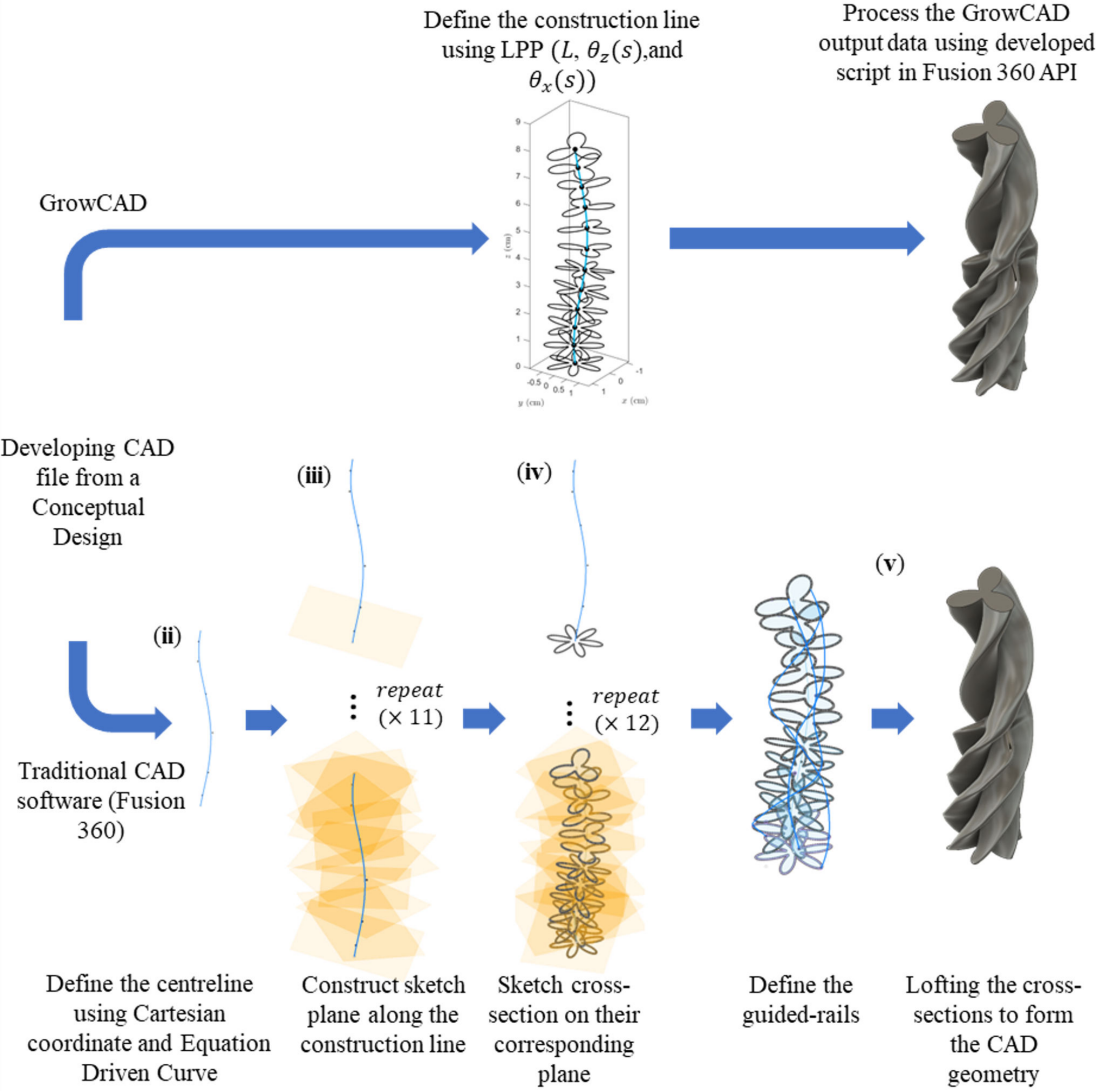
Comparison in computational process to develop a CAD file using GrowCAD^TM^ and Fusion 360.

A qualitative comparison was performed between the computational process of constructing a geometry in GrowCAD^TM^ with that in traditional CAD software, in this instance Fusion 360. Figure 5 represents the difference in methodology. Complex geometry or curves in CAD can be created manually through a range of various techniques, including creating and manipulating surfaces, freeform editing of solids, lofts and sweeps and integrating haptic devices to manipulate 3D models directly [40,41]. This study is specific to curved geometries, relative to a construction line and using traditional CAD construction methods; such a geometry would be represented using a centre line and/or a series of cross-sections. The sweep function maintains a constant cross-section along a defined path, and the loft function can connect cross-sections with various sizes and shapes.

#### 5.1.1. Computer-aided design

Complex geometrical shapes with various sizes and shapes can be created in Fusion 360 using the following steps (with respect to figure 5):

(i) Install the ‘Equation Driven Curve’ Plug-in [39].
(ii) Define the construction line using the ‘Equation Driven Curve’ function and the appropriate coordinate system (cylindrical, polar or cartesian). A Cartesian example is given in equations (5.1)–(5.3).
(iii) Use the ‘Plane Along the Path’ function to create normal planes along the construction line for individual cross-sections.
(iv) Create the individual cross-section using the ‘Equation Driven Curve’ function on its corresponding sketch plane.
(v) Connect the cross-section to form a 3D CAD model using the loft function (guided or non-guided).

(5.1) $$x\left(s\right)=\frac{4}{\pi }\sin\left(\frac{\pi }{18}\right)\sin\left(2\pi s\right),$$

(5.2) $$y\left(s\right)=\frac{1}{\pi }\left(-4\sin\left(\frac{\pi }{18}\right)cos\left(2\pi s\right)+4\sin\left(\frac{\pi }{18}\right)\right),$$

(5.3) $$z\left(s\right)=8\cos\left(\frac{\pi }{18}\right)s,$$

where *s* varies from 0 to 1.

In addition, the sketch may be required to rotate or tilt about an axis of rotation. The choice of the loft function, i.e. the guided and non-guided, depends on the complexity of the cross-sections. Using a guided loft function for complex geometry with asymmetric cross-sections allows shape control by affecting how it travels between profiles and leads to the precise formation of the CAD file. This necessitates the creation of guided rails, which are series of spline curves which intersect with each cross-section. The steps required to define the guided rails and these are outlined in appendix A.2. Finally, whilst manually constructing this geometry in CAD (Fusion 360) there is no associated manufacturing analysis, unless it is further synchronized with CAM.

#### 5.1.2. GrowCAD^TM^

GrowCAD^TM^ creates the same geometry, through a much simplified process, predominately due to the LPP definition of the construction line denoted as L, $\theta_{z}(s)$, and $\theta_{x}(s)$ (equations (5.4)–(5.6) and P3 in table 2). The cross-sections are associated with the centre line, as outlined in §3.2. The manufacturing constraints, represent an additional functionality to the CAD methodology outlined in §5.3.1, are analysed with respect to the overall geometry, as outlined in §3.3.

(5.4) L=8 (cm),

(5.5) $$\theta_{z}\left(s\right)=10^{\circ },$$

(5.6) $$\theta_{x}\left(s\right)=360s^{\circ },$$

where *s* varies from 0 to 1.

Figure 5 demonstrates the shape fidelity of GrowCAD^TM^ equivalent geometry. Direct comparison of equations (5.1) to (5.3) with (5.4) to (5.6) demonstrates the relative simplicity of the LPP algebraic definition, integrated into the GrowCAD^TM^ framework.

### 5.2. Manufacturability

The functionality of GrowCAD^TM^ software was evaluated through the P1 experiments using the geometrical constraints of overhang angle and wall thickness, which significantly affect the manufacturability of the designed parts. The parameters used for the construction of these geometries are presented in table 2. The equation-driven approach of GrowCAD^TM^ permits an infinite number of geometric designs, and as such, only a representative selection can be presented within the paper.

The constructs generated were compared with those created using Fusion 360, and its manufacturability was assessed by: minimum solid thickness (measured in Fusion 360) and overhang angle (measured using Netfabb Premium 2021 (Autodesk, San Francisco, CA, USA)). The results are shown in table 3. For each, a representative image is presented. Binary (pass/ fail) results indicate whether the geometry was manufacturable. This experiment will validate whether the software can identify geometries that breach the manufacturing constraints.

**Table 3.** Assessing the functionality of GrowCAD^TM^ through the geometrical constraints of overhang angle and wall thickness.

	GrowCAD^TM^ construction feedback	GrowCAD^TM^ geometry	digital CAD validation (Fusion 360)	digital manufacturability validation (Netfabb)
P1-1-1	pass	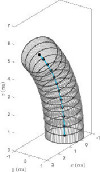	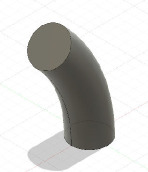	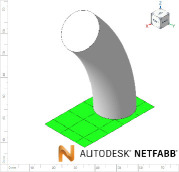
P1-1-2	fail (overhang angle exceeds 45°)	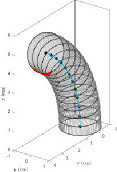	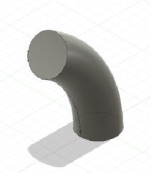	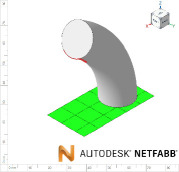
P1-1-3	pass	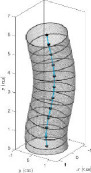	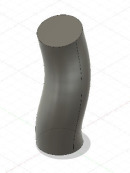	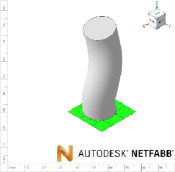
P1-1-4	fail (overhang angle exceeds 45°)	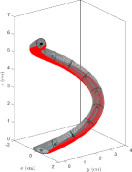	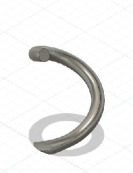	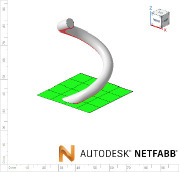
P1-2-1	pass	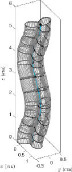	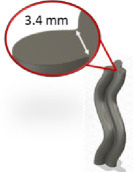	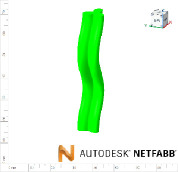
P1-2-2	fail (outer surface pinching too thinly at the centre of cross-sections)	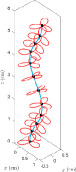	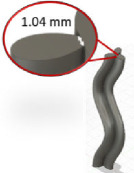	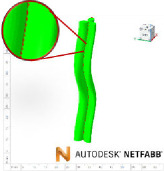

### 5.3. End-to-end compatibility

**Figure 6.**
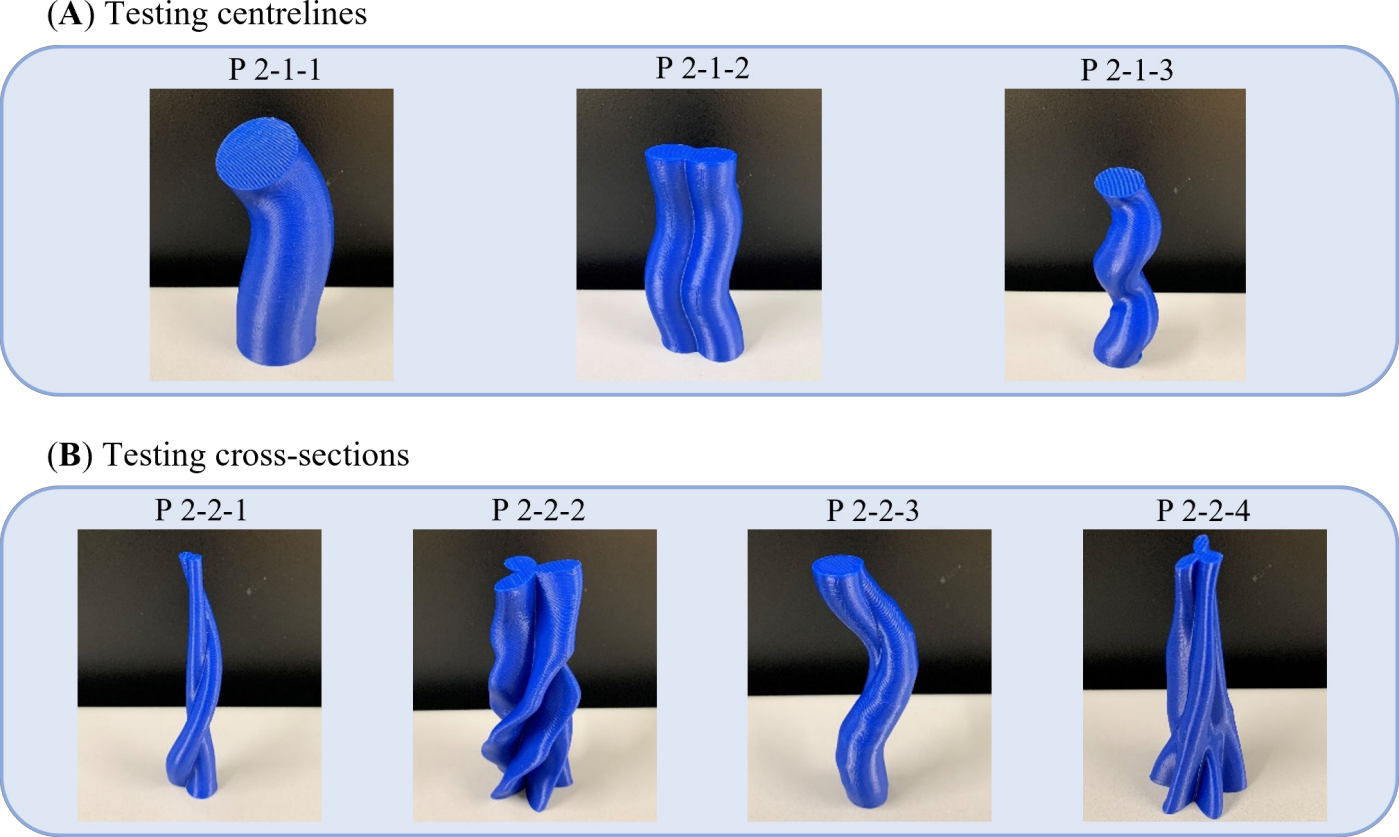
Three-dimensional printed representative models for experiment P2 to design and manufacture various models through using different (A) construction line: off-axis single helix (P2-1-1), out-of-plane 2D wave (P2-1-2), on-axis double helix construction line (P2-1-3), and (B) cross-section by varying its size (P2-2-1), shape (P2-2-2), rotation (P2-2-3), size, shape and rotation simultaneously (P2-2-4).

The final experiment, P2, has been designed to validate the practical compatibility of GrowCAD^TM^ with CAD, CAM and finally FDM 3D printing for the designed structure and assess its performance within the manufacturing context. The details for individual models are listed in table 2. While the algebraic representation, will enable an infinite number of geometric designs, in this study, variables associated with the centre line and cross-sections were validated. The parts were printed three times (*n* = 3). Each build contained multiple prints, and each repeat was printed in a separate build. All builds were successful. Figure 6 represents the manufactured parts produced by incorporating various types of construction line and cross-sections. Due to the volume of results, one representative image of the physical results is given. The results will be assessed in a binary manner (success or not). Additional images are shown in appendix B.

## 6. Discussion

While research and commercial methods exist for the optimization of structures in the context of advanced manufacturing techniques, the processes which underpin manual definition of parametrically defined geometry in CAD have existed in a relatively unchanged format for decades. There have been huge developments in terms of creating complex geometry (which may be formed using AM), by integrating, for example, AI [42] or field definitions [43]. However, these techniques and many other automated approaches do not enable the engineer to understand how to create manually defined, editable and manufacturable geometry. The research presented in this study, used the intersection of mathematical biology and engineering to develop a mathematical representation of geometry in a way that more innately reflects the process of AM. The algebraic definition of a curved construction line in 3D and a closed surface around the line supports the intuition behind layer-by-layer AM construction, in the same way that centre line-fitted mathematical descriptions of slender geometries support the modelling of plant root growth.

The aim of this research was to develop a CAD-compatible mathematical design approach for 3D printing, to enable more efficient generation of manufacturable curved geometry around a centre line. In essence, a manual approach (increasing socioeconomic accessibility) to enable the construction of organic structures, more commonly associated with automated techniques such as topology optimization. The novel research contribution presented in this article is as follows: the implementation of the centre line LPP coordinate system (equations (3.1)–(3.3)) mathematically derived in full in [38], assessment of manufacturing constraints of the geometry (interpolation of the LPP associated cross-sections) relative to the TNB or XYZ alignment of LPP associated cross-sections, a Python-enabled automated interface with CAD. It should be noted that the mathematical description of the cross-sections (table 2) in isolation, while not common in engineering applications, is not considered a novel research contribution. The focus of the implementation and validation was to confirm the shape fidelity, increased efficiency and functionality of the approach. The simplicity of the approach was compared against an equivalent CAD process. The digital function was assessed, using defined manufacturing constraints, against pre-existing software. The end-to-end functionality, in terms of compatibility with CAD, CAM and FDM 3D printing, was assessed experimentally.

### 6.1. Shape fidelity and efficiency

To demonstrate the efficiency of the proposed framework, the method used to construct an arbitrary framework using GrowCAD^TM^ was compared with traditional CAD methodology (figure 5). The equations required to define the geometry using Cartesian coordinates in CAD are shown in equations (5.1)–(5.3) and in GrowCAD^TM^ in equations (5.4)–(5.6). The difference in mathematical complexity is not easily quantifiable; however, qualitatively and intuitively comparing the two sets of equations shows that the LPP method is a drastically simpler representation. Industrial engineers are rarely specialists in mathematics and the XYZ definition of the construction line would likely be beyond the scope of many. When verbally describing a curve, one would use language referring to its length and relative angle [38]. The mathematical description of equations (5.1)–(5.3) does not have an intuitive meaning in human language. In contrast, equations (5.4)–(5.6) reflect how one would verbally describe the behaviour of a curve: length 8 cm (equation (5.4)), with a constant angle of 10° relative to the vertical (equation (5.5)) while twisting around the *z*-axis by one revolution (equation (5.6)). Thus, something easily described in natural language and is translatable to mathematics using the LPP framework.

The robust nature of the mathematical computation, in high-curvature regions, is ensured by the discretization (128 steps between neighbouring construction points) and the numerical integration method utilized to compute the coordinates of all discretization points (fourth-order Runge–Kutta). Theoretically, it is possible to make the variation in curvature increasingly extreme; however, any incremental errors can be smoothed by increasing the (user-defined) number of construction points.

Human intuition is inherently subjective; what is intuitive to one person may not be to another. With respect to how engineers learn and contextualize information regarding the manufacturing constraints associated with DfAM, there have been several studies which demonstrate the importance of learning by doing, for example, through problem-based learning [44,45]. While in this study, no time difference was measured between the two processes, it is hypothesized that the technique would lead to an increased in efficiency and speed of modelling complex geometries and also knowledge-translation back to the designer. Future research would require academic and industrial designers to test the GrowCAD^TM^ technique against a range of commercial CAD software, including participants with extensive experience in complex surface modelling (as described in §2.1).

### 6.2. Manufacturability

The novelty in this research, stems from the implementation of user-defined manufacturing constraints (falling more broadly under the heading of manufacturability) into the description of geometry. These are non-trivial to combine while also retaining parametric editability, within the design process. Assessing manufacturing constraints during process planning is common-place using widely available CAM for extrusion-based AM, such as UltiMaker Cura (Utrecht, The Netherlands) or through more platform-specific techniques. However, once the design process has reached CAM, the parametric editability has been lost. Therefore, to make significant edits to the geometry with respect to manufacturability will require repetition of the CAD through to CAM process. While other approaches exist to assess manufacturability, to the best of the authors’ knowledge, these are undertaken once the process has been discretized and/or automated, i.e. parametric editability has been lost. Jayakody *et al*. [46] propose a vector analysis of boundary overhang angle in the context of process planning for support-free multi-axis printing [46]. Commercial optimization techniques, including topology optimization and generative design, often include geometric manufacturability constraints and/or assessment. In comparison with these automated approaches, it is unlikely that the framework presented in this research would offer a more efficient method, because it is a manual technique. However, the benefit of GrowCAD^TM^ over ‘black-box’ software is the ability to upskill the designer (§6.1), which offers benefits throughout the entire process of design, including conceptual design and the design to manufacturing interface. Open-source software approaches also exist, for example Z88Arion^®^ [47], which uses finite spheres to define manufacturing constraints [48,49].

Table 3 demonstrates that the framework is able to detect contradictions in manufacturing constraints of overhang angle and wall thickness, which is equivalent to manual measurements of wall thickness in Fusion 360 (CAD) and automated measurements of overhang angle in Netfabb (CAM). In GrowCAD^TM^, the analysis of wall thickness takes an (automated) measurement perpendicular to the boundary of the cross-section. It is noted that there are instances where the overhang angle warning is very conservative, for example, P1-1-4. The overhang constraint is tested by finding an intersection between the surface and the base of a cone extended from each point in the designed part (§3.1.1). When the surface curvature is high, there are instances where it does not intersect the base of the cone even though the apex point is supported, leading to a false-positive ‘overhang exceeded’ warning. In experiment P1-2-2 (table 3), the algorithm can detect the centre thickness, but it cannot detect the minimum thickness at the outer lobes of the 3pt star. This is because the lobes of the 3pt star do not demonstrate less than the minimum wall thickness, in a direction perpendicular to curve. However, the wall thickness may be less than the minimum, in a direction not perpendicular to the curve. In this study, the lobes of the star were captured by the parallel wall layers, as opposed to the infill and therefore the printing was a success. The results demonstrate the efficacy of the approach, within the definition of thickness given in this study. Generalization beyond this study should be undertaken with caution and in the context of the mathematics.

The mathematical representation of the geometry presented here operates independently of manufacturing and materials. This mirrors how traditional CAD interfaces with CAM and 3D printing; and as such, represents its compatibility with CAD. The approach incorporates user-defined manufacturing constraints, which can be customized for wall thickness and overhang angle associated with any three-axis AM techniques, which have the same geometric limitations. To realize this compatibility, the overhang and wall thickness constraints associated with the platform, material(s) and parameters would need to be tailored through empirical testing or academic literature. Whilst the manufacturability analysis presented in this study requires discretization of the geometry, the advantage that GrowCAD^TM^ offers above traditional CAD is that the manufacturing constraints are highlighted during the construction of the geometry and in the context of the algebraic definition.

### 6.3. End-to-end compatibility

Figure 6 (and additional images in appendix B) demonstrates the printability of the geometry, which is mathematically defined through the LPP coordinate system in table 2. Table 2 explores distinct geometries of the construction line (single helix, double helix and 2D wave) and cross-sections (size, shape, rotation and orientation). All prints were successful; no prints failed due to overhangs that were too large or wall thicknesses that were too thin. It can be concluded that for the geometries described in table 2, the manufacturing constraints were successfully considered during the design using the GrowCAD^TM^ framework, following through to the CAM and then 3D printing.

The GrowCAD^TM^ framework offers an opportunity to explore the design space of complex user-defined curves within the constraints of manufacturability, a significant improvement on current approaches to constructing geometry. As such, the user can explore the geometric opportunities and constraints of AM simultaneously, increasing both the creativity and underlying knowledge of the designer. The absence of the ‘black-box’ approach means that creating the geometry and then, through use, retaining the knowledge used to create the geometry are two separate outcomes. There is very little research demonstrating how to measure design creativity. Prabhu *et al*. [50] demonstrated teaching both opportunistic and restrictive DfAM, generated designs with a higher ‘technical goodness’, as opposed to restrictive DfAM alone [50]. The authors hypothesize that the intuitive nature of the mathematical description of the geometry, i.e. equations (5.4)–(5.6), through repeated use, will enable increased knowledge of the user. However, this will require further research to confirm.

### 6.4. Limitations, developments and translation

**Figure 7.**
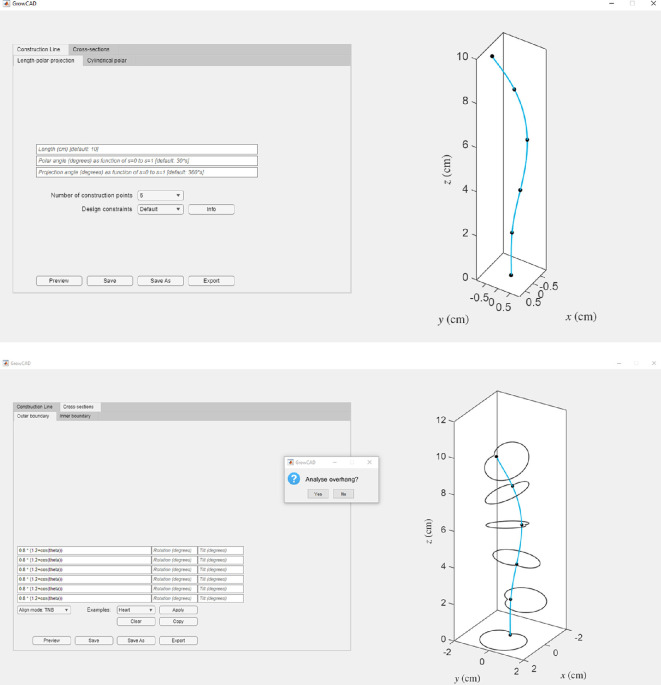
Two screenshots of the GrowCAD^TM^ user interface. Top: the ‘Construction Line’ screen, using the novel LPP system (§3.1) to specify a curve in 3D space. The values shown, and the construction line and points generated, are built-in defaults, while the input fields allow user customizations. Bottom: the ‘Cross-sections’ screen, allowing custom designs of non-axisymmetric cross-sections at the construction points under either the TNB or the XYZ alignment mode, with user-specified rotation and tilt angles (§3.2). The dropdown menu ‘Examples’ contains mathematical expressions for various shapes (‘hearts’ are shown here). An analysis of wall thickness is carried out on the cross-sectional boundaries (§3.3.1) and proceeding to ‘analyse overhang’ through the pop-up dialogue initiates an analysis of overhang angles on the extrapolated surface (§3.3.2), which produces results such as those in table 3.

The approach detailed in this research has been computationally and experimentally validated for a range of inputs. Even though the chosen inputs are representative of common geometries, it must be acknowledged that not all of the infinite number of possible inputs can be tested. The results reported in this study are evaluated relative to the inputs listed in the design for experiments (table 2). The case study presented in this paper was specifically compared with one mainstream, commercially available CAD programme, to evaluate the efficiency of the approach. A custom Python script was written to interface between GrowCAD^TM^ and Fusion 360. The API (outlined fully in §4.1) functions as follows: input the point cloud of the cross-sections, define the sketch plane, create a spline from the point cloud, extract rails through the cross-sections and along the length of the structure, perform a loft. In principle, such a technique could be used to interface GrowCAD^TM^ with any CAD software. A limitation of the study is the user interface, which was enabled by MATLAB and shown in figure 7. Considering future translation of the technique, while there are built-in equation examples to guide the user upon first use, a more user-friendly interface and/or tutorial would be required. However, this development represents another significant translational research/development and is beyond the scope of this TRL1-3 engineering–mathematics research.

The optimization of process parameters in 3D printing is not a trivial task; it requires repeated empirical testing and is only valid to a specific combination of material and process [4]. As such, the manufacturing constraints of the Creality Ender-3 were not optimized in this study and assumed to be arbitrary but well-acknowledged geometric thresholds (specific to FDM). As with all commercial software or research approaches to support DfAM, the success and/or limitations of future use will be specific to the functional application, material, process and parameters.

In the context of longer-term industrial or clinical application, the implementation of GrowCAD^TM^ shall be outlined. As mentioned above, the methodology could be integrated into a bespoke and more accessible user interface. The main novelty of this approach stems from the LPP coordinate system, which in its current presentation could be directly integrated into a commercial CAD interface. This would be compatible with pre-existing parametric equation modelling and enhance its functionality and suitability to modelling for AM. This would enable print-direction-dependent generation of complex curves within the design of a larger part—through importing the geometry or direct generation within a part/assembly. As discussed above, the process can be tailored to any manufacturing constraints, and the mathematics are unlimited to scale, and therefore it is widely adaptable to any material and process. A limitation of this study is the absence of an application-based case study and a comparison to an alternative DfAM approach, which will be addressed in future work. To enable the visualization of an end-use application, GrowCAD^TM^ would be well suited to design (for example) complex manifolds or lattice structures.

The additional benefit that GrowCAD^TM^ offers (while unvalidated in this study) is to upskill the engineer through use of the approach—which will firstly reduce knowledge gaps in education [15] and take a step towards the future-proofing skills against the rapidly changing landscape of AM technologies. This could offer further positive outcomes, in terms of utilizing design to address complex global challenges; inclusive design requires a wide variety of perspectives to create socially and environmentally sustainable solutions. Using GrowCAD^TM^ as a training tool, to enable stakeholder participants to understand the geometric capability of an AM platform, could offer more efficient brainstorming during the conceptual design phase.

## 7. Conclusions

The premise of this research was to overcome the challenges associated with designing additively manufacturable geometry in traditional CAD. Key requirements were identified through the academic literature: a digital framework to create geometry within the capabilities of AM, the ability to tailor this to specific materials and platform, compatibility with parametrically editable CAD, and the capacity to increase the users’ knowledge and/or upskill the user through use. A bioinspired approach, facilitated through mathematical biology, was used to address this problem, drawing an analogy between the layer-by-layer formation of a structure during AM and the growth of a plant root. The research presents an algebraic equation-based definition of a construction line where the mathematical complexity is significantly reduced compared with the CAD equivalent. Three measures were used to validate the implementation:

— Qualitative analysis of shape fidelity and efficiency, compared with a traditional CAD construction.
— The ability to detect geometry which falls outside of the bounds of the (user-defined) manufacturing constraints, compared with commercial analysis tools.
— The end-to-end functionality with CAD, CAM and FDM 3D printing and associated manufacturing constraints.

In addition, compared with current commercial and automated approaches, the simplicity and intuitive nature of the technique may offer an increased user understanding of the mathematical definition of 3D curves and surfaces.

## Figures and Tables

**Figure 8.**
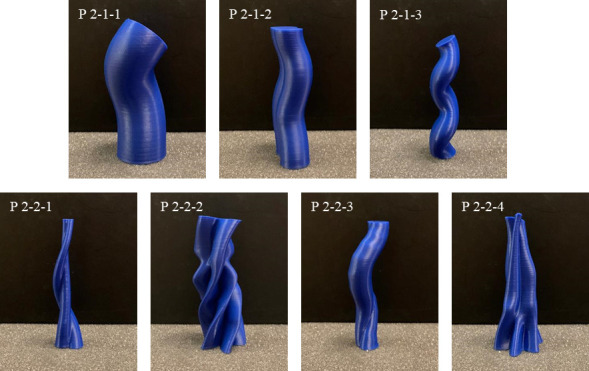
Additional perspectives of 3D printed representative models for experiment P2 to design and manufacture various models through using different (top) construction line: off-axis single helix (P2-1-1), out-of-plane 2D wave (P2-1-2), on-axis double helix construction line (P2-1-3), and (bottom) cross-section by varying its size (P2-2-1), shape (P2-2-2), rotation (P2-2-3), size, shape and rotation simultaneously (P2-2-4).

## Data Availability

The datasets supporting this article have been uploaded as part of the electronic supplementary material (S1, S2 and S3 [51]).

## Footnotes

^1^
GrowCAD.m is freely available upon any reasonable request made to the corresponding author.

## Appendix A

### A.1.

See figure 7.

### A.2.

The steps required to define guided rails are as follows:

— Split the cross-sections based on the required number of guided rails.
— Add points on those locations on each cross-section.
— Connect the corresponding points from each cross-section using the spline function to form individual guided rails.

## Appendix B

See figure 8.
